# Inferring the structures of signaling motifs from paired dynamic traces of single cells

**DOI:** 10.1371/journal.pcbi.1008657

**Published:** 2021-02-04

**Authors:** Raymond A. Haggerty, Jeremy E. Purvis

**Affiliations:** 1 Department of Genetics, University of North Carolina, Chapel Hill, North Carolina, United States of America; 2 Computational Medicine Program, University of North Carolina, Chapel Hill, North Carolina, United States of America; 3 Curriculum for Bioinformatics and Computational Biology, University of North Carolina, Chapel Hill, North Carolina, United States of America; 4 Lineberger Comprehensive Cancer Center, University of North Carolina, Chapel Hill, North Carolina, United States of America; Northeastern University, UNITED STATES

## Abstract

Individual cells show variability in their signaling dynamics that often correlates with phenotypic responses, indicating that cell-to-cell variability is not merely noise but can have functional consequences. Based on this observation, we reasoned that cell-to-cell variability under the same treatment condition could be explained in part by a single signaling motif that maps different upstream signals into a corresponding set of downstream responses. If this assumption holds, then repeated measurements of upstream and downstream signaling dynamics in a population of cells could provide information about the underlying signaling motif for a given pathway, even when no prior knowledge of that motif exists. To test these two hypotheses, we developed a computer algorithm called MISC (Motif Inference from Single Cells) that infers the underlying signaling motif from paired time-series measurements from individual cells. When applied to measurements of transcription factor and reporter gene expression in the yeast stress response, MISC predicted signaling motifs that were consistent with previous mechanistic models of transcription. The ability to detect the underlying mechanism became less certain when a cell’s upstream signal was randomly paired with another cell’s downstream response, demonstrating how averaging time-series measurements across a population obscures information about the underlying signaling mechanism. In some cases, motif predictions improved as more cells were added to the analysis. These results provide evidence that mechanistic information about cellular signaling networks can be systematically extracted from the dynamical patterns of single cells.

## Author summary

Cells use molecular signaling networks to translate dynamically changing stimuli into appropriate downstream responses. Specialized network structures, or motifs, allow cells to properly decode a variety of temporal input signals. In this paper, we present an algorithm that infers signaling motifs from multiple examples of an upstream signal paired with its downstream response in a population of single cells. We compare the predictive power of single-cell versus averaged time-series traces and the incremental benefit of adding more single-cell traces to the algorithm. We use this approach to understand how yeast respond to environmental stresses.

## Introduction

**Fig 1:**
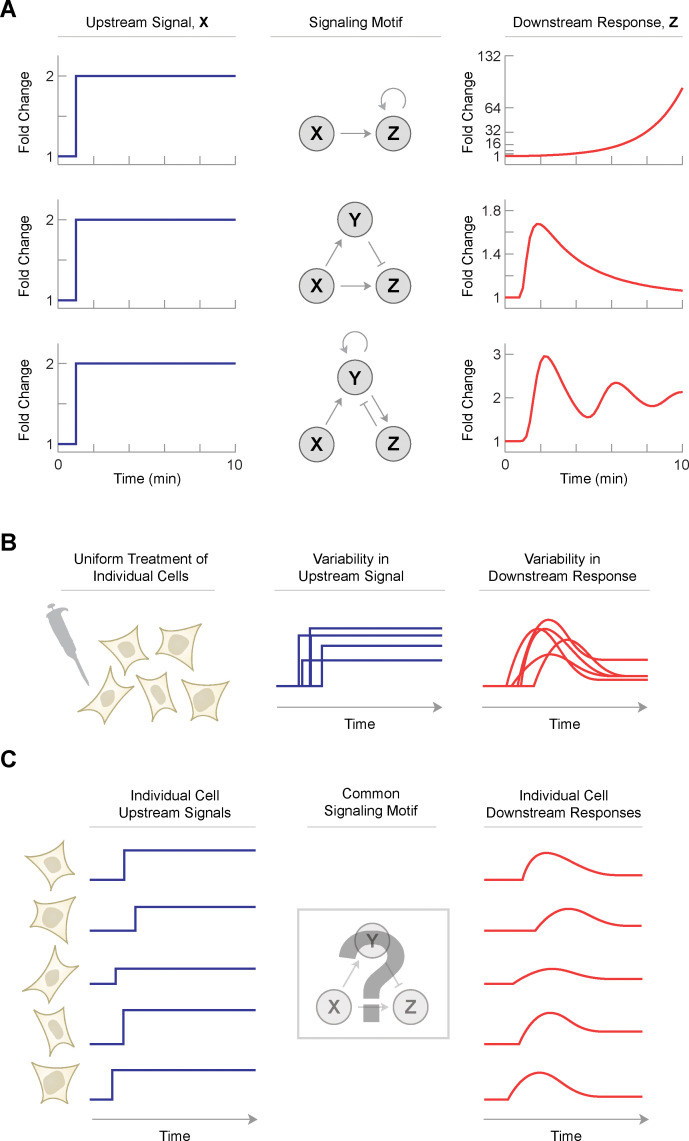
Signaling motifs determine how upstream signals are converted into downstream responses. (A) The same upstream signal, X, can produce different downstream responses, Z, depending on the signaling motif. Positive feedback leads to rapid amplification of Z following a delay in its induction. An incoherent feedforward loop (IFFL) allows Z to adapt to changes in X by first activating then dampening the downstream response. Coupled positive and negative feedback can lead to oscillations of Z. Signaling motifs often involve an intermediate signaling factor, Y, that is necessary to achieve the appropriate downstream response. Ordinary differential equations for each signaling motif are provided in the S1 Text. (B) In response to a given stimulus, individual cells show heterogeneous signaling patterns. For many cellular signaling pathways, the variability of the upstream signal, X, is correlated with the downstream response, Z. (C) Hypothetical model for a common signaling motif that explains the correlation between upstream signaling and downstream responses. Differences in upstream signal are mapped onto the downstream response. Under this model, it may be possible to infer the underlying structure by observing many examples of the upstream and downstream signaling patterns.

Cells interpret complex temporal patterns of molecular signals to execute appropriate downstream responses such as changes in gene expression or cell fate [1–3]. The molecular factors that participate in these signaling networks are often organized into specialized network structures, or motifs, that carry out a specific signal-processing function [4–6]. For example, a positive feedback loop can facilitate strong and irreversible responses to an upstream signal such as the commitment to cell division [7]. A negative feedback loop, such as the metabolic response to changes in blood insulin, allows cells to adapt to different levels of an upstream signal [8,9] or to filter signaling noise [10]. More complex network motifs, such as coupled positive and negative feedback, can lead to oscillations [11,12]. Here, we use the term “upstream signals” to refer to the inputs that initiate signaling in a particular pathway. Examples of an upstream signal include the activity or expression level of a receptor, kinase, or second messenger. These signals are decoded by specialized motifs into “downstream responses” such as changes in gene expression or epigenetic state (Fig 1A). Understanding the signaling motifs that decode upstream signals into downstream responses is a major goal of systems biology because these mechanisms define the dynamic relationships among signaling components and provide quantitative predictions about the cellular response to pharmacological intervention [13].

Interestingly, not all cells respond to the same upstream signal in an identical way. Previous studies have shown that individual cells show considerable heterogeneity in their dynamic responses to the same input stimulus (Fig 1B) [14,15]. Stimulation with epidermal growth factor (EGF), for example, leads to differences in extracellular signal-related kinase (ERK2) activity [16,17]. Similarly, uniform induction of DNA damage leads to heterogeneous patterns of p53 dynamics [18]. In many cases, these differences in signaling dynamics are correlated with distinct cell fate decisions. For example, the temporal pattern of cyclin-dependent kinase 2 (CDK2) activity after mitosis—although variable from cell to cell—predicts whether a cell will proceed directly into S-phase or spend additional time in G1 [19]. Similarly, sustained ERK2 activity following serum addition indicates that cells will enter S-phase [16]. Such correlations suggest that differences in upstream signaling patterns play an important role in driving cells toward specific downstream responses.

These collective observations suggest a testable hypothesis: if differences between individual cells have predictable effects on downstream responses, then perhaps all cells in that population share a common signaling motif that consistently interprets each cell’s unique signaling dynamics (Fig 1C). In other words, upstream signals are consistently “mapped” to downstream responses depending on their unique temporal dynamics. If such a single mechanism exists in all cells, then a second hypothesis follows: by using many examples of the upstream signals and downstream responses, it may be possible to infer the underlying signaling motif without any prior knowledge of its structure. That is, single-cell signaling patterns gathered under the same experimental conditions could reveal the structure of the molecular mechanism that produced those very patterns.

Here, we test these two hypotheses by analyzing paired sets of upstream and downstream signals in single cells. We focus specifically on inferring the signaling motifs that control the stress response of budding yeast [20,21]. We introduce a computational approach called Motif Inference from Single Cells (MISC) that determines the mechanistic relationship between measurements of two fluorescent reporters in the same cell. MISC exploits the heterogeneity among identically treated cells by finding one or more signaling motifs that best explain the relationship between the paired upstream signals and downstream responses across all cells in a population. Although many excellent network inference tools have been established [22,23], many of them work on a different type of data, employ averages, or do not allow for hidden nodes. We believe this to be the first tool that infers dynamic networks from paired time-series traces from single cells and allows for hidden nodes. Our results provide evidence that single-cell dynamics contains information that reveals the underlying signaling mechanisms that produced them.

## Results

### Signaling motifs convert upstream signals into downstream responses

**Fig 2:**
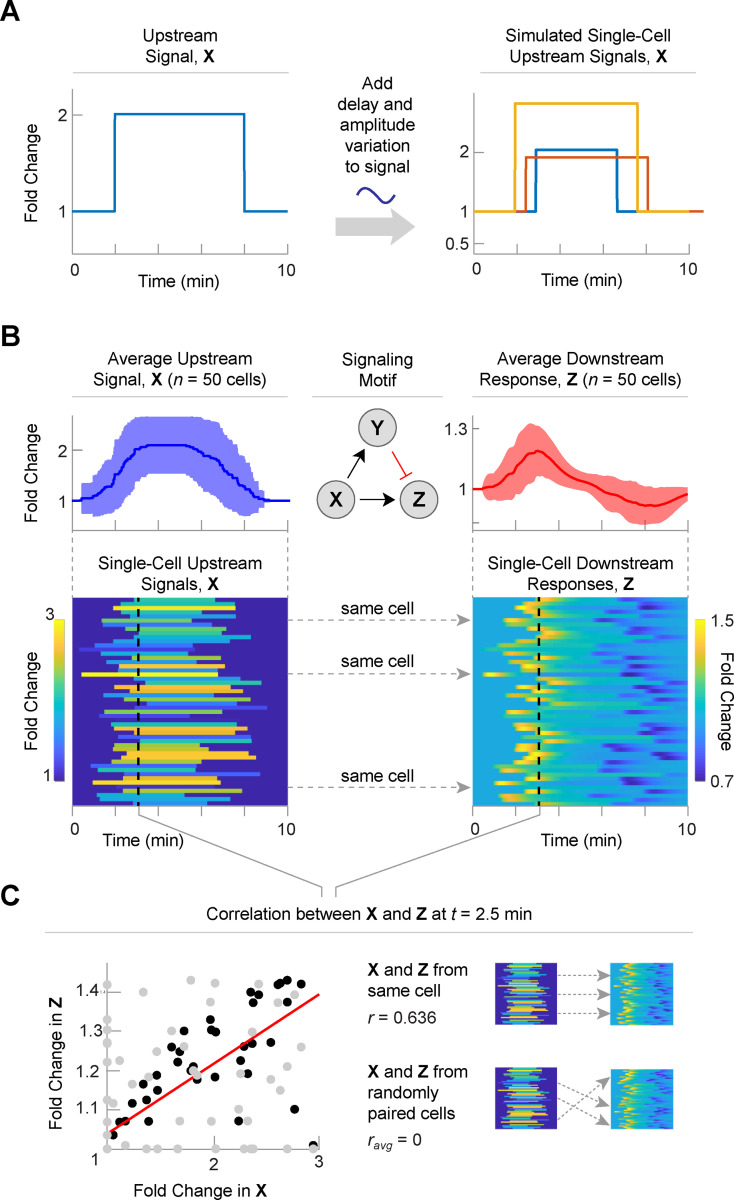
A single signaling motif can explain the correlation between upstream signals and downstream responses in single cells. (A) Experimentally measured values for cell-to-cell variation in signal height and delay were used to generate a large number of simulated signaling patterns. Each trace represents a unique upstream signal from an individual cell. (B) Average (*top*) and single-cell (*bottom*) signaling patterns for upstream signals (*left*) and downstream responses (*right*) for 50 simulated cells. While X is generated using the approach in panel A, the downstream responses are calculated using the signaling motif (middle). Here, Z if produced by the incoherent feed forward loop. A single signaling motif translates 50 upstream signals into 50 downstream responses. Each row corresponds to an individual cell. Color represents the fold change. (C) There is a correlation between the upstream and downstream signals that is not present if the cells are shuffled. Black dots are from paired cells and grey dots are from shuffled cells. The red line is a correlation line for the paired cells. The unpaired cells do not have any correlation.

We hypothesized that cell-to-cell variation in an upstream signal can be decoded by a common signaling motif that produces an accompanying set of downstream responses. Under this model, the original source of variability in the upstream signal is not considered. Instead, we focus on the mechanistic relationship between the upstream signal and the downstream response. To explore this, we first considered how variation in an upstream signal might be propagated by a single signaling motif. We chose a simple upstream signaling pattern represented by a rapid 5-fold increase above basal levels after a 2 min delay and a return to basal levels after 6 minutes. To simulate realistic cell-to-cell variability in this signaling pattern, we allowed each cell to deviate from the average behavior in its amplitude and delay time (see Materials and Methods and S1 Text for details on how simulated single cell data was generated). This procedure allowed us to generate an arbitrarily large number of unique upstream signaling patterns that closely resembled experimental measurements from individual cells (Fig 2A).

We next generated 50 individual upstream signals, X, and asked how these simulated signaling patterns could be decoded by a common signaling motif (Fig 2B). For an initial analysis, we selected the incoherent feedforward loop (IFFL) as the signaling motif because of its well-characterized behavior [24,25]. An IFFL involves three signaling factors (X, Y, and Z). X positively regulates Y and Z, and Y negatively regulates Z. The mechanism is represented as a set of ordinary differential equations (ODEs) that allow calculation of the downstream response given a particular upstream signal. We used the Hill-Langmuir equations to represent positive and negative interactions among X, Y, and Z but derived new expressions for the right-hand side of the ODEs to accommodate fold-change representations of X, Y, and Z rather than absolute quantities (S1 Text and S1 Tutorial).

As expected, the average response to the step increase in X is a transient increase in Z that returns to basal levels as the accumulation of Y dampens the increase in Z and when the signal in X disappears, the cells overcorrect and drop below basal, and then return to basal levels. At the single-cell level, each cell showed a slightly varying downstream response in accordance with the upstream signal. When the upstream signal for each cell was plotted against its downstream response at 2.5 min, these differences showed a moderately positive correlation (Fig 2C, black dots). This analysis is analogous to performing immunohistochemistry to detect levels of X and Z across a large population of cells at a fixed time point. Importantly, this correlation was destroyed when the upstream signal of a given cell was randomly paired with the downstream response of a different cell (Fig 2C, gray dots). Thus, for this analysis, the correlation between upstream signals and downstream responses among individual cells reflects a mechanistic relationship between these two signaling factors.

These results show how a single signaling motif can lead to different downstream responses by propagating variation in an upstream signal. Importantly, the signaling motif is deterministic: it does not invoke any stochastic processes to generate different downstream responses. Although it has been widely observed that individual cells have different molecular constitutions that can lead to variation in signal transduction, this exercise demonstrates how cell-to-cell differences in input signals may be mapped to unique downstream responses by virtue of a single signaling motif that is common to all cells.

### Inferring the signaling motif from simulated single-cell dynamics

**Fig 3:**
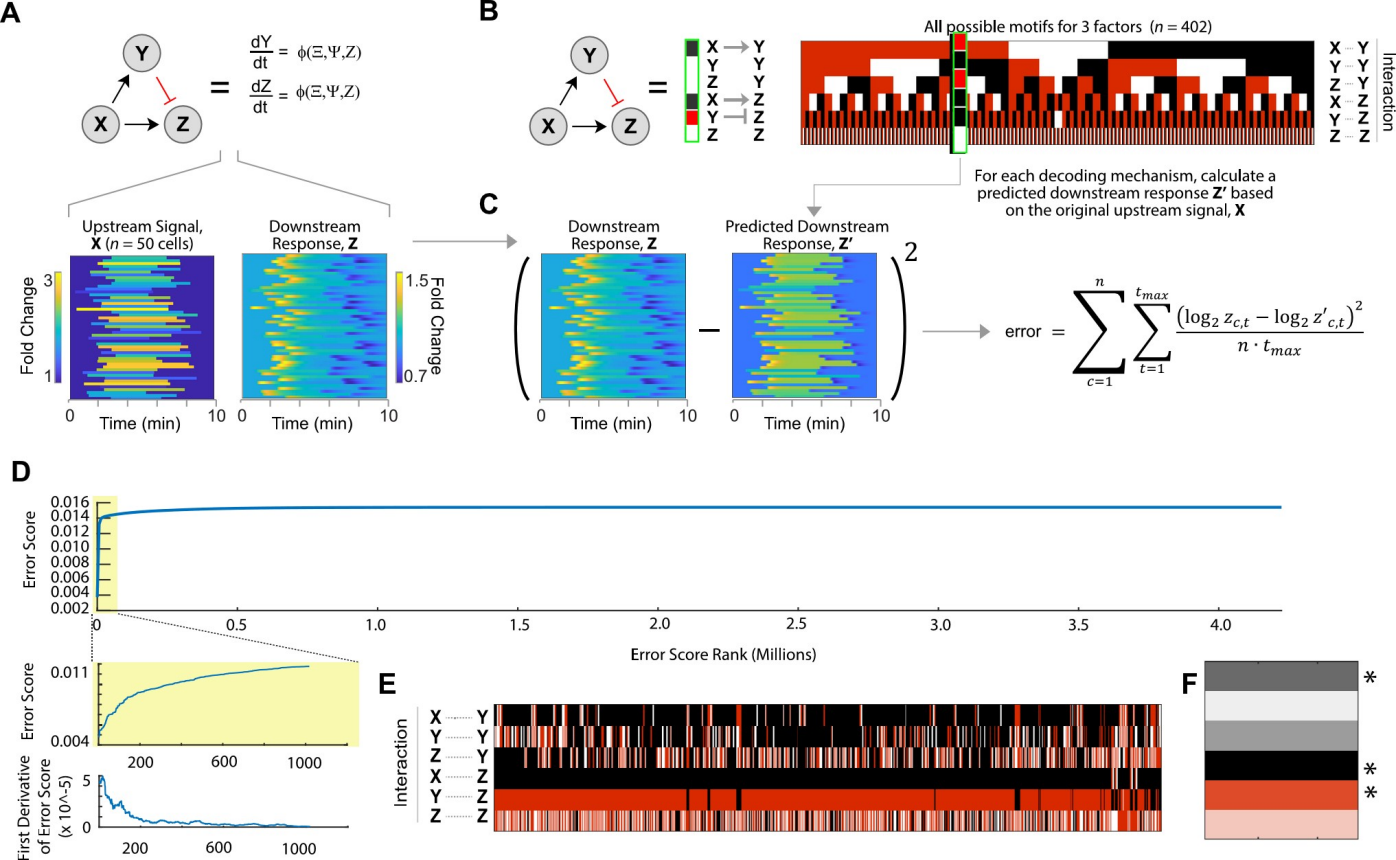
Inferring the underlying signaling motif from simulated single-cell dynamics. (A) An incoherent feedforward loop (IFFL) signaling motif was used to generate a synthetic data set for 50 single cells. Individual upstream signals X were generated as shown in Fig 2A. Each signal was then subjected to the IFFL signaling motif using ODEs to calculate a unique downstream response Z for each cell (see S1 Text). The resulting X and Z pairs for all cells were then analyzed to infer the underlying signaling motif. (B) Heat map representation of motif structures for all possible 3-factor signaling motifs. (*left*) A conventional representation of motif structure consisting of circles and arrows can be represented by (*right*) a single column of shaded rectangles in which each rectangle denotes a specific interaction within the motif: black, positive regulation; red, negative regulation; white, no regulation. The heat map representation for the IFFL is shown next to the conventional diagram. (Right) All 402 possible unique motif structures are shown for a 3-component network (X, Y, and Z). (C) To infer the mechanism underlying X and Z, a separate downstream response, Z’, is calculated for each possible parameterized signaling motif and compared to the original downstream response, Z. Each motif is assigned an error score that reflects the average difference in fold change between Z and Z’ per cell per unit time. (D) Distribution of error scores. The error scores are distributed such that they approach the asymptote of the worst possible score (i.e., the output of an unconnected graph). The top graphs are defined as those before the “elbow” of the graph and are determined by looking at the first derivative. (E) The top-scoring motifs. The links for the incoherent feed forward loop, as well as its simpler sub-motif structures, are overrepresented among the best-performing motifs. (F) Heat map representation of the consensus motif. Asterisks indicate a greater than 50% confidence in that link being correct. Darker colors mean more confidence and lighter colors mean less confidence. The distance from the consensus motif to the known motif was XX which has a p-value of 9.95x105.

For many signaling pathways, the signaling motif that converts upstream signals into downstream responses is unknown [16,19,26]. We developed a computer algorithm called Motif Inference from Single Cells, or MISC, that infers signaling motifs from measured single-cell dynamics. Before applying the algorithm to an experimental data set, we first tested MISC on the synthetic data set generated by a known signaling motif described above. Fig 3A shows the upstream signaling dynamics and downstream responses for 50 simulated cells using an IFFL as the signaling motif. To infer the signaling motif in an unbiased way, we considered all possible structures of signaling motifs that could explain the relationship between X and Z, including mechanisms that involve a potential intermediate signaling factor, Y. Although it is possible to include more than one unknown factor in our search, we limited our search to networks of 3 signaling factors as proof of principle for several reasons. These reasons include: First, there are practical limitations in the number of non-overlapping fluorophores that can be measured simultaneously by live imaging in order to validate predictions. Second, there are practical limitations in computation time involved in increasing the number of intermediate signaling nodes. Third, there is a predominance of well-characterized biological motifs that contain 3 or fewer factors [5,27] Fourth, 3-factor motifs can be combined to create more complicated networks; Lastly, the additional node, Y, can represent a subnetwork of one or more factors. We did not consider feedback to X because X is explicitly provided to the algorithm and reports on its own feedback.

The complete set of signaling motifs can be succinctly represented as a heat map (Fig 3B). In this visual format, the regulatory relationships between each pair of factors is represented by a black or red box indicating positive or negative regulation, respectively. After eliminating redundant or trivial motif structures (e.g., motif containing only Y), there are 402 possible signaling motifs for a 3-factor signaling motif that allows distinct positive and negative interactions. Each mechanism was converted to set of ODEs that were used to calculate the downstream response Z for a given upstream signal X (see S1 Text). We fixed the sign (positive or negative) of all regulatory interactions in the mechanism and used 40,000 randomly selected, bounded, parameter sets to parameterize each of the ODEs. We used a Monte Carlo approach rather than fitting parameters because we want to determine which motifs are robust to random parameterization. Next, each of the dynamic traces for this calculated response, Z’, were compared to the original downstream response to calculate an error for each signaling motif (Fig 3C). Using this approach, all signaling motif and parameter combinations receive an error score. Comparing these errors provides the bases for inferring the underlying signaling motif: predicted responses that best matched the original downstream response were hypothesized to be more structurally similar to the original (i.e., unknown) motif.

Fig 3D shows the errors for all randomly parameterized motifs, ranked in ascending order of error when the output of each motif was compared to the synthetic data. We found that the distribution of ranked error scores rapidly approached an asymptote of the worst possible score, which represents the output of a motif with no signaling output (i.e., a flat line at Z’ = 1). This score represents the performance of a motif with no predictive power. The vast majority of the motifs produced errors that were close to the error asymptote. To identify well-performing motifs in a principled way, we then looked for the “elbow” of the graph in which the curve began to flatten out and the errors approached the asymptote. Algorithmically, we do this by looking at the first derivative and finding the point where the slope falls below a certain percentage of the maximum slope (Fig 3D, *inset*). By systematically varying this percentage and evaluating the accuracy of motif predictions, we found that a cutoff of 10% was optimal. This is the location where the slope begins to level off (See S1 Text).

After separating the motif-parameter combinations into well-performing and poorly-performing motifs, we look among the well-performing motifs for the most overrepresented motifs. We do this by taking the most common link for each connection and calculating how confident we are that is the correct link by looking at how often that connection appears and then calculating a confidence score based on the percentage of times it appears. When we did this, we arrived at a motif that was similar to the IFFL. In fact, all of the connections that were greater than 50% confidence were exactly the IFFL. Statistical significance was calculated by comparing the confidence score to a sample of random graphs with random connections and confidences. In this manner, MISC was used to successfully infer the correct signaling motif for a synthetic data set in which the underlying motif structure was already known.

**Fig 4:**
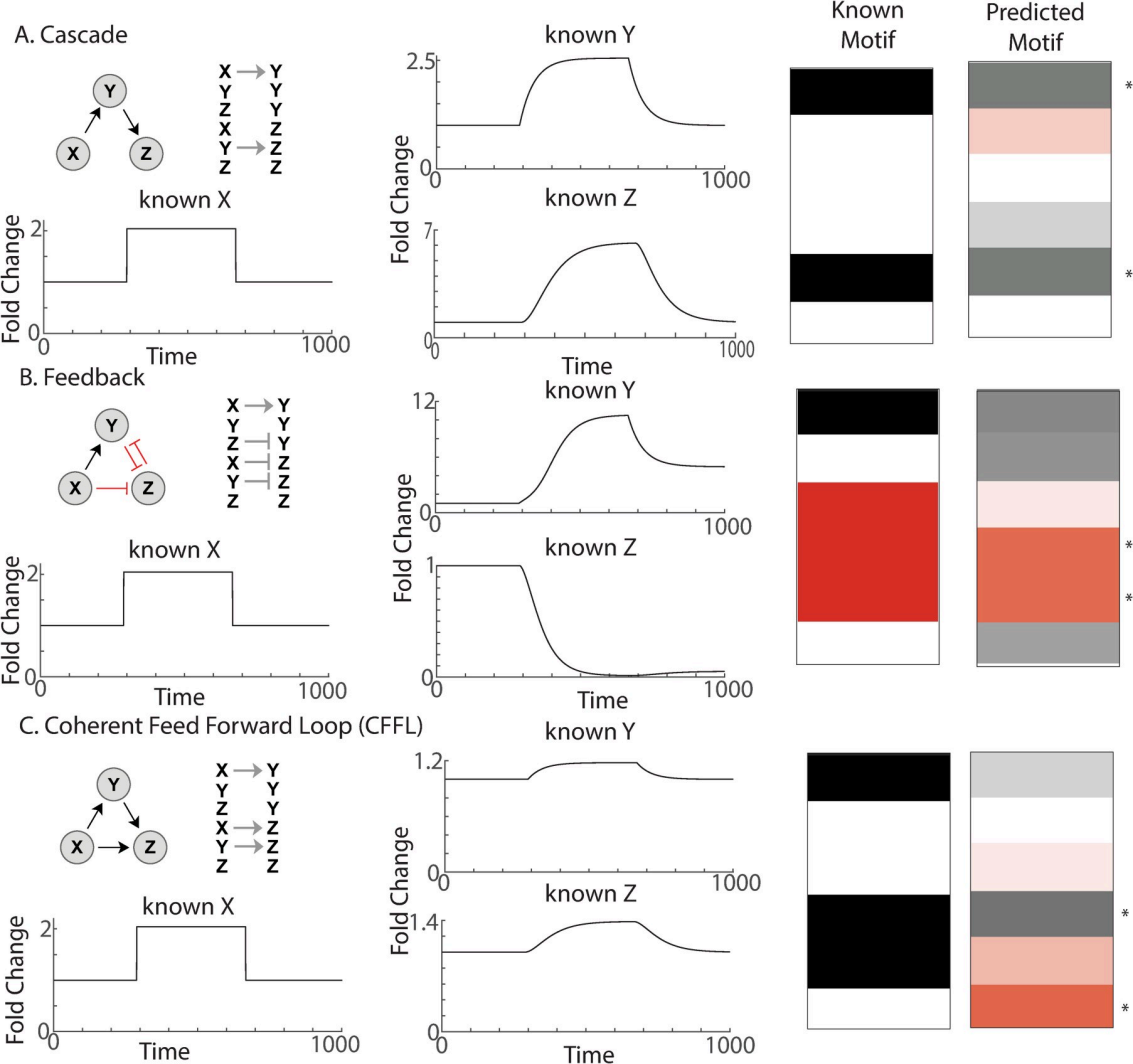
Validation of MISC with additional biological motifs. (A) Cascade motif. The cascade motif creates a delay in the output, Z. Upper left, ball and stick model and arrow representation of the motif. Lower Left and Middle, an example of the motif. Right, Known and predicted motif. Asterisks indicate greater than 50% confidence in that connection. (B) Feedback motif. The feedback motif creates a persistence in the response of Z. Upper left, ball and stick model and arrow representation of the motif. Lower Left and Middle, an example of the motif. Right, Known and predicted motif. Asterisks indicate greater than 50% confidence in that connection. (C) Coherent Feed Forward Loop (CFFL) motif. The CFFL creates a delay in the onset of Z, but no delay in the offset. Upper left, ball and stick model and arrow representation of the motif. Lower Left and Middle, an example of the motif. Right, Known and predicted motif. Asterisks indicate greater than 50% confidence in that connection.

We further validated MISC on three more additional, well-characterized biological motifs: the cascade, feedback, and coherent feed forward loop [27]. The cascade (Fig 4A) is a motif where X indirectly activates Z through Y. It creates a delay in the output, Z. MISC predicted a consensus motif which was very similar to the correct motif. All of the connections with confidence over 50% were the correct connections. The entire motif was closer to the correct motif than by chance (*P* = 0.000623). The feedback (Fig 4B) is a motif which creates a persistence in the response of Z. MISC was able to correctly identify the X to Z and Y to Z connections as being important with over 50% confidence. It also predicted a positive connection from X to Y and a negative connection from Z to Y. The Coherent Feed Forward Loop (Fig 4C) creates a delay in the onset of Z, but no delay in the offset. Although the exact motif was not inferred, the results suggest a different “functionally equivalent” motif that produces the same behavior.

### Different yeast stress response pathways show use of the same signaling motif

**Fig 5:**
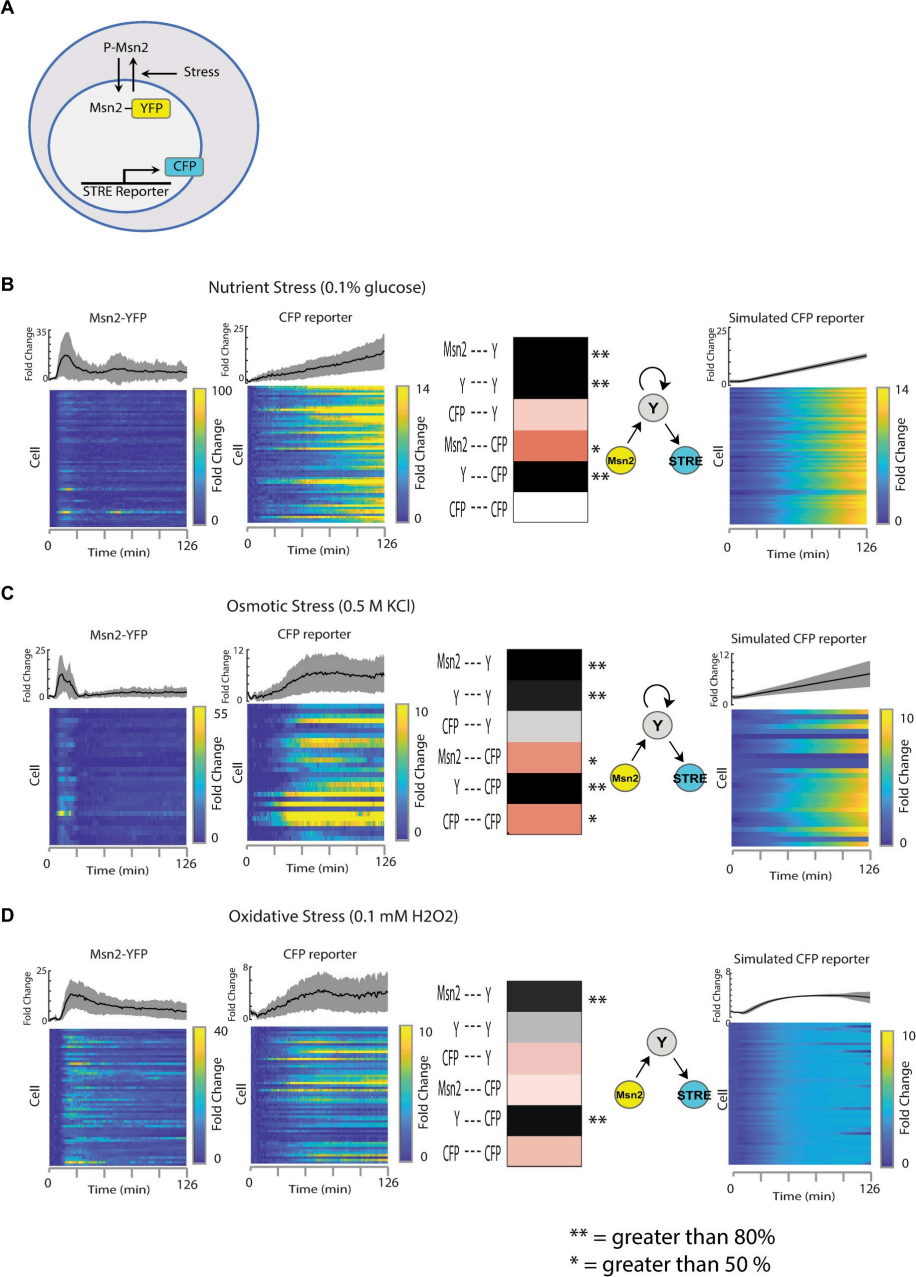
Motif inference for the yeast stress response pathway under different environmental perturbations. (A) Schematic of the system showing the fluorescent reporter system used. See ref. 21 for experimental details. Experimental data were used with author permission. (B) Oxidative Stress. 58 cells were treated with 0.1 mM H_2_O_2_. Far left, heatmap of the individual cell’s Msn2 activation, and a plot of the mean and standard deviation of the cells. Left middle, STRE response as reported by the CFP reporter. Right middle, heatmap and ball and stick representations of the significant sub-motifs. Single asterisks represent 50% confidence and double asterisks represent 80% confidence. Displayed as ball and stick representations are the 80% confidence connections. Far right, Simulated STRE response of the top preforming sub-motif. (C) Nutrient Stress. 60 cells were treated with a 0.1% glucose media. Far left, heatmap of the individual cell’s Msn2 activation, and a plot of the mean and standard deviation of the cells. Left middle, STRE response as reported by the CFP reporter. Right middle, heatmap and ball and stick representations of the significant sub-motifs. Single asterisks represent 50% confidence and double asterisks represent 80% confidence. Displayed as ball and stick representations are the 80% confidence connections. Far right, Simulated STRE response of the top preforming sub-motif. (D) Osmotic Stress. 28 cells were treated with 0.5 M KCl. Far left, heatmap of the individual cell’s Msn2 activation, and a plot of the mean and standard deviation of the cells. Left middle, STRE response as reported by the CFP reporter. Right middle, heatmap and ball and stick representations of the significant sub-motifs. Single asterisks represent 50% confidence and double asterisks represent 80% confidence. Displayed as ball and stick representations are the 80% confidence connections. Far right, Simulated STRE response of the top preforming sub-motif.

We next applied MISC to determine the underlying signaling motifs from experimental data. We focused on the transcriptional response to environmental stress in budding yeast, which has been studied extensively using time-lapse fluorescence microscopy [21,28,29]. To predict the signaling motifs at work in the yeast stress response, we used previously published single-cell data in which two signaling activities were measured in the same cell over time [21]. Here, the upstream signal X is the transcriptional activity of the transcription factor MSN2. Upon stress, MSN2 is translocated to the nucleus where it promotes transcription of multiple target genes (Fig 5A). The downstream response Z is a fluorescent protein driven by several DNA stress response elements (STREs). Thus, X and Z represent the signaling pathway that transmits upstream stress signals to the downstream expression of target genes that allow yeast to appropriately cope with stress.

We first examined the response to nutrient stress with 0.1% sugar in 60 single cells (Fig 5B). When the activity of all cells was averaged, MSN2 showed a transient 18-fold increase followed by a return to near-basal levels. As reported previously, many cells showed a prominent pulse of MSN2 nuclear localization followed by smaller, more sporadic pulses. The averaged downstream response of STRE gene expression showed a gradual, almost linear, increase in expression. Individual cells showed considerable variability in downstream response, with some cells displaying very little changes in expression. To predict the underlying signaling motif for this stress response, we applied the MISC algorithm to the full single-cell data set. The consensus motif had several strongly confident connections over 80%. These connections made a cascade motif with additional feedback on Y from itself. This predicted motif structures are similar to the previously published two-step model of transcription, in which the STRE promoter must first transition from an inactive to active state before productive transcription can begin [21].

We next considered the yeast response to osmotic stress, using a smaller data set consisting of 28 single cells that were treated with 0.5 M KCl (Fig 5C). Under this condition, MSN2 levels showed a transient 12-fold increase in nuclear localization followed by a near-perfect adaptation to basal levels, leveling off around 3-fold. The downstream response showed a sudden increase in expression around 20 minutes after treatment that reached sustained 7-fold levels within the first 60 minutes. As with sugar stress, individual cells showed varying behavior and sporadic pulses of MSN2 activity. When we applied MISC, we got another consensus motif with several strongly confident connections over 80%. As in the case with the nutrient stress, these connections made a cascade motif with additional feedback on Y from itself.

Finally, we considered the yeast stress response to oxidative stress using a data set of 57 individual cells (Fig 5D). Under this experimental condition, MSN2 levels showed a more prolonged response to stress, reaching 14-fold change that adapted more slowly than either salt or sugar stress. The downstream response of the STRE reporter showed a biphasic response, characterized by a gradual increase that peaked at 1 h, followed by a transient decrease and subsequent increase in expression. In this case, the strongly confident, over 80% predicted connections made the cascade motif. Notably, the consensus motifs for all three stresses were similar, suggesting that even though the stresses and the responses were different, the core machinery in place to decode the stresses is the same [21].

### Single-cell dynamics contain information about the underlying motif structure that is not present in the population-averaged response

**Fig 6:**
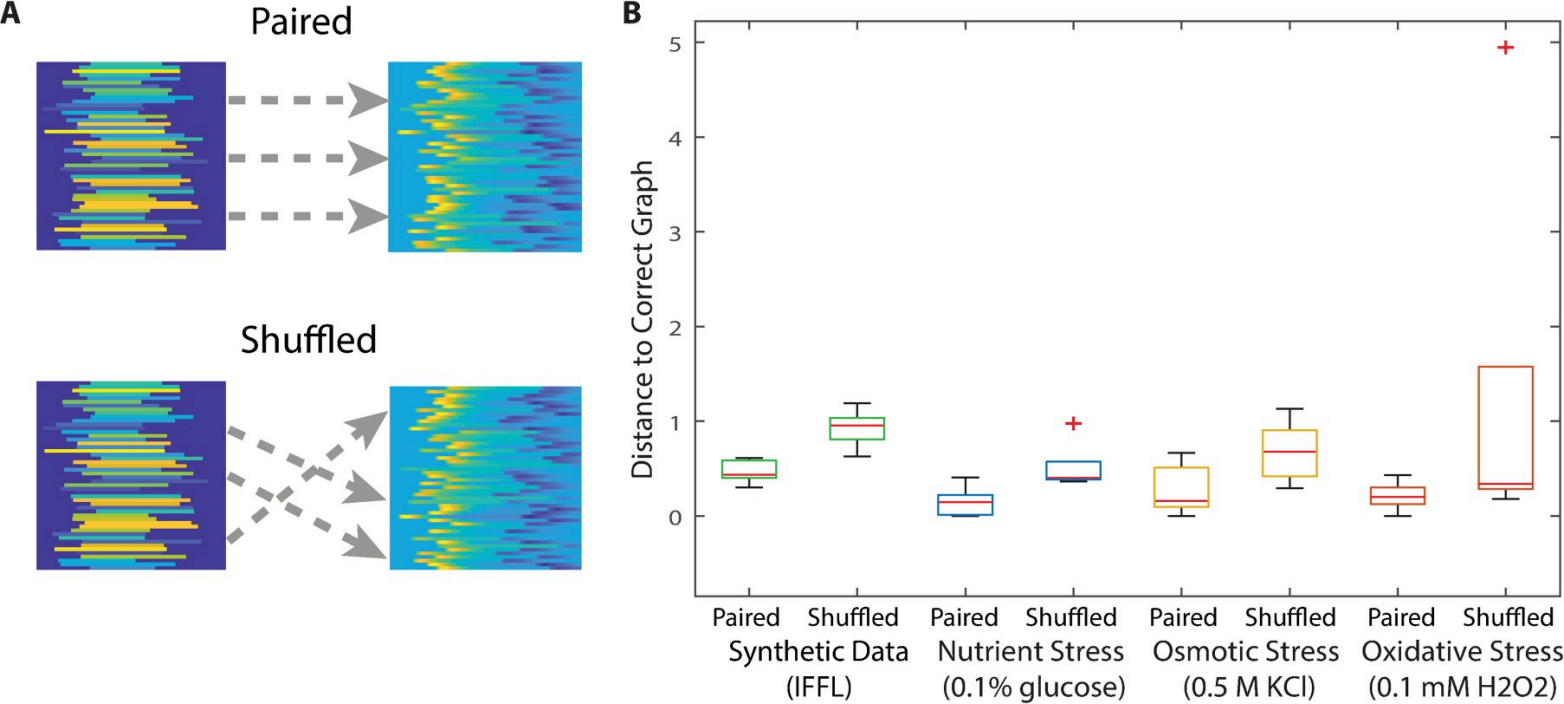
Single-cell dynamics can provide improved certainty or accuracy in motif prediction than population-averaged data. (A) In order to evaluate motif prediction based on single-cell versus population-averaged time-series traces, we performed MISC on pairs of time-series traces that were either correctly paired (*top*) or randomly shuffled (*bottom*). Note that in both cases the average time series trajectory is the same. (B) Shuffled versus paired predictions for the IFFL and yeast stresses. Ten replicates were used for each condition. Note that MISC produces variability in predictions even for paired traces since parameter values on the motifs are randomly chosen.

Previous studies have predicted motif structures based on a single time series [25]. It is unclear what additional value, if any, is provided by multiple single-cell measurements. To answer this question, we performed MISC on the same stress response data but permuted the data so that the upstream signal X for a given cell was randomly paired with the downstream response Z of another cell from the same experiment. Importantly, the population-averaged response remains the same under this perturbation, but the relationship between MSN2 and STRE at the level of individual cells is lost (Fig 6A).

As proof of principal, we tested this permutation strategy on the synthetic single-cell data generated by the IFFL (Figs 2 and 3). Predictions from matched cells were consistently better at predicting the correct structure (*P* = 0.00038) than randomly paired cells, which predicted a motif further away (*P* = 0.0097) (Fig 6B). We repeated this on the natural stresses and found that they followed the same trends of being better at predicting the correct structure (taken by considering the >80% confidence connections) when outputs were properly paired with inputs than when they were shuffled. Taken together, these results show that at least part of the predictive power of MISC comes from the observed relationships between the dynamics of X and Z in individual cells. This result suggests that information about the underlying signaling motif can be extracted from paired time series measurements in single cells.

**Fig 7:**
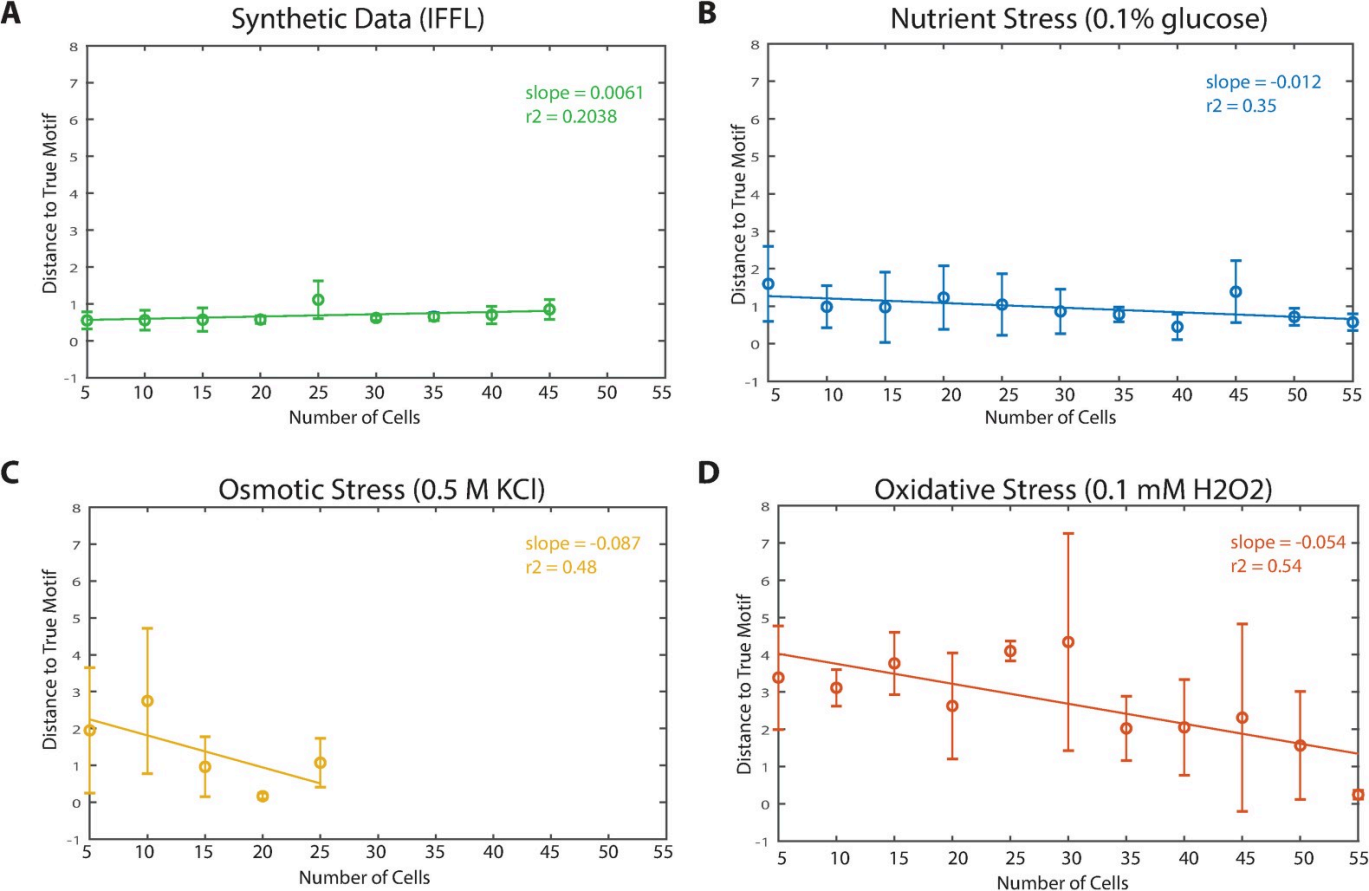
Additional single-cell measurement can provide increasingly better predictions about the underlying signaling motif. MISC was performed on an increasing number of paired single-cell measurements and the accuracy of the prediction (based on the distance to the true motif or the motif calculated by the 80% threshold shown in Fig 5) was calculated. (A) Synthetic Data, (B) Nutrient Stress, (C) Osmotic Stress, (D) Oxidative Stress. The Synthetic Data did not decrease, but all of the natural stress predictions decreased in percentage as the number of cells increased.

We next asked whether adding additional cells to the MISC analysis could improve its predictive power. To address this question, we calculated the percentage of time the correct sub-motif appeared in the top-performing motifs as a function of the number of cells included in the analysis. As a benchmark, we subsampled the synthetic IFFL—starting with five cells—and then incrementally adding more cells until we reached the total number of cells collected under each condition. For each of these subsampled sets of cells, we ran the MISC algorithm and calculated the distance to the correct motif (as determined by using all cells). We performed five replicates per sample size, choosing a random subset of cells for each replicate. The IFFL showed no gain in predictive power as more cells were added to the analysis (Fig 7A). This result was expected because, in the synthetic data set, each cell’s downstream response is deterministically generated by its upstream response. The absence of intrinsic noise in the synthetic data renders each cell a perfect predictor of the underlying motif structure [30,31].

We then performed this analysis again on the experimentally-determined stress data, using the top performing sub-motif that encapsulates all higher-ranking sub-motifs. Under glucose limitation, MISC showed only slight improvement with the addition of more cells. (Fig 7B). Under both salt and oxidative stress, however, we observed an increase in predictive accuracy as we increased the number of single cell traces subjected to MISC (Fig 7C and 7D). Given the limited number of cells gathered experimentally, it is unclear at what number of cells the predictive accuracy of MISC would have leveled off. Nevertheless, these results demonstrate that each pair of time-series traces from a single cell adds incremental value to the quality of the motif prediction.

## Discussion

It is commonly stated in the field that single-cell measurements contain information that is obscured by population-averaged methods such as western blotting and quantitative PCR [32–37]. Indeed, single-cell approaches have revealed a staggering degree of heterogeneity among individual cells in terms of gene expression and protein dynamics. Here, we exploit those cell-to-cell differences by asking whether they arise from the same underlying generative process. Simulations show that we can predict a signaling motif based on paired sets of dynamic traces. As these types of measurements become more common in single cell studies [38–40], MISC may prove helpful in inferring the underlying motif structures.

We found that predictions were strengthened by considering the coupled relationships between two signals in individual cells, and that adding additional cells to the analysis can improve, in some cases, the certainty and accuracy of motif predictions. A similar strategy was used to discover signaling proteins that affect cell motility [41]. By screening a library of fluorescent fusion proteins for proteins that correlate with cell movement, this apparent “noise” in protein expression was used to identify novel regulators of motility.

Many biological network inference approaches have focused on predicting the topology of a pathway using static or population-averaged data that does not consider the single-cell dynamics of the system. Inference based on dynamical data was previously applied to global profiling data, in an effort to identify temporal transition nodes and edges [42]. MISC offers a novel inference strategy that examines a smaller scope of a biological system and aims to infer the mechanistic relationships among the measured components using single-cell dynamics. MISC allows nonlinear interactions between signaling factors. In addition, MISC allows the functional form of the governing equations to be modified to better suit various systems (S1 Text).

Another strength of MISC is the ability to infer components of the system that were not directly measured. Although it may be possible to measure the upstream signal and downstream response for a given signaling pathway, signaling motifs often involve additional molecular intermediates or regulatory relationships that are unmeasured or unknown. For example, it may be possible to measure the activity of a transcription factor along with target gene expression in single cells [16,20,43]. However, additional factors, such as cofactors or chromatin modifying complexes, may modulate the downstream response [44]. Yet, knowledge of these signaling motifs is necessary to quantify how the upstream signal is decoded to produce the observed downstream responses. We present MISC as a tool that can hypothesize unknown signaling intermediates based on the dynamical behaviors of interacting signaling factors. As with other inference methods, the motif predictions made my MISC can be experimentally tested and used to make future predictions about the cell’s behavior.

Finally, we show how increasing the number of single-cell traces improves the certainty and accuracy of motif prediction. Future studies are warranted to determine whether there is a most economical number of single-cell traces to collect under a certain experimental condition in order to identify the underlying signaling network that generated the traces. This is a practical consideration in many live-cell studies, in which accurate quantification of traces can been laborious and time-consuming, and for which there are often no best-practices for how many cells should be collected and analyzed. In addition, it is unclear how extrinsic noise affects the identifiability of network motifs, or whether a lack of identifiability due to extrinsic noise can be overcome by an increase in sample size. Further investigation is needed to more fully characterize and quantify the practical limits of motif inference from time-lapse imaging measurements.

## Materials and methods

MATLAB scripts for all simulations, calculations, and figures are provided as Supplementary Information and are available at https://figshare.com/projects/Inferring_the_structures_of_signaling_motifs_from_paired_dynamic_traces_of_single_cells/73275

## Supporting information

S1 Text Supplemental Information. (DOCX)

S1 Tutorial Tutorial for how to run MISC. (PDF)

## References

[1] Purvis JE, Lahav G. Encoding and decoding cellular information through signaling dynamics. Cell. 2013;152(5):945–56. 10.1016/j.cell.2013.02.005 23452846 PMC3707615

[2] Kholodenko BN, Hancock JF, Kolch W. Signalling ballet in space and time. Nature reviews. 2010;11(6):414–26. Epub 2010/05/25. nrm2901 [pii] PubMed Central PMCID: PMC2977972. 10.1038/nrm2901 20495582 PMC2977972

[3] Levine JH, Lin Y, Elowitz MB. Functional roles of pulsing in genetic circuits. Science. 2013;342(6163):1193–200. Epub 2013/12/07. 10.1126/science.1239999 . 24311681 PMC4100686

[4] Bhalla US, Iyengar R. Emergent properties of networks of biological signaling pathways. Science. 1999;283(5400):381–7. 10.1126/science.283.5400.381 . 9888852

[5] Alon U. Network motifs: theory and experimental approaches. Nat Rev Genet. 2007;8(6):450–61. Epub 2007/05/19. nrg2102 [pii] 10.1038/nrg2102. 10.1038/nrg2102 . 17510665

[6] Behar M, Hoffmann A. Understanding the temporal codes of intra-cellular signals. Curr Opin Genet Dev. 2010;20(6):684–93. Epub 2010/10/20. S0959-437X(10)00166-8 [pii] 10.1016/j.gde.2010.09.007. 10.1016/j.gde.2010.09.007 20956081 PMC2982931

[7] Trunnell NB, Poon AC, Kim SY, Ferrell JE Jr. Ultrasensitivity in the Regulation of Cdc25C by Cdk1. Molecular cell. 2011;41(3):263–74. 10.1016/j.molcel.2011.01.012. 10.1016/j.molcel.2011.01.012 21292159 PMC3060667

[8] Kubota H, Noguchi R, Toyoshima Y, Ozaki Y, Uda S, Watanabe K, et al Temporal Coding of Insulin Action through Multiplexing of the AKT Pathway. Molecular cell. 2012;46(6):820–32. Epub 2012/05/29. S1097-2765(12)00340-1 [pii] 10.1016/j.molcel.2012.04.018. 10.1016/j.molcel.2012.04.018 . 22633957

[9] Goentoro L, Kirschner MW. Evidence that fold-change, and not absolute level, of beta-catenin dictates Wnt signaling. Molecular cell. 2009;36(5):872–84. 10.1016/j.molcel.2009.11.017 20005849 PMC2921914

[10] Becskei A, Serrano L. Engineering stability in gene networks by autoregulation. Nature. 2000;405(6786):590–3. 10.1038/35014651 . 10850721

[11] Lahav G, Rosenfeld N, Sigal A, Geva-Zatorsky N, Levine AJ, Elowitz MB, et al Dynamics of the p53-Mdm2 feedback loop in individual cells. Nat Genet. 2004;36(2):147–50. Epub 2004/01/20. 10.1038/ng1293 . 14730303

[12] Nelson DE, Ihekwaba AE, Elliott M, Johnson JR, Gibney CA, Foreman BE, et al Oscillations in NF-kappaB signaling control the dynamics of gene expression. Science. 2004;306(5696):704–8. Epub 2004/10/23. 306/5696/704 [pii] 10.1126/science.1099962. 10.1126/science.1099962 . 15499023

[13] Behar M, Barken D, Werner SL, Hoffmann A. The dynamics of signaling as a pharmacological target. Cell. 2013;155(2):448–61. 10.1016/j.cell.2013.09.018 24120141 PMC3856316

[14] Davis DM, Purvis JE. Computational analysis of signaling patterns in single cells. Seminars in Cell and Developmental Biology. 2014 10.1016/j.semcdb.2014.09.015 25263011 PMC4339661

[15] Regot S, Hughey JJ, Bajar BT, Carrasco S, Covert MW. High-sensitivity measurements of multiple kinase activities in live single cells. Cell. 2014;157(7):1724–34. Epub 2014/06/21. 10.1016/j.cell.2014.04.039 24949979 PMC4097317

[16] Albeck JG, Mills GB, Brugge JS. Frequency-Modulated Pulses of ERK Activity Transmit Quantitative Proliferation Signals. Molecular cell. 2013;49(2):249–61. Epub 2012/12/12. 10.1016/j.molcel.2012.11.002 . 23219535 PMC4151532

[17] Cohen-Saidon C, Cohen AA, Sigal A, Liron Y, Alon U. Dynamics and variability of ERK2 response to EGF in individual living cells. Molecular cell. 2009;36(5):885–93. Epub 2009/12/17. S1097-2765(09)00866-1 [pii]10.1016/j.molcel.2009.11.025. 10.1016/j.molcel.2009.11.025 . 20005850

[18] Geva-Zatorsky N, Rosenfeld N, Itzkovitz S, Milo R, Sigal A, Dekel E, et al Oscillations and variability in the p53 system. Mol Syst Biol. 2006;2:2006 0033 Epub 2006/06/15. msb4100068 [pii]10.1038/msb4100068. 10.1038/msb4100068 16773083 PMC1681500

[19] Spencer SL, Cappell SD, Tsai FC, Overton KW, Wang CL, Meyer T. The proliferation-quiescence decision is controlled by a bifurcation in CDK2 activity at mitotic exit. Cell. 2013;155(2):369–83. 10.1016/j.cell.2013.08.062. 10.1016/j.cell.2013.08.062 24075009 PMC4001917

[20] Hansen AS O’Shea EK. Promoter decoding of transcription factor dynamics involves a trade-off between noise and control of gene expression. Mol Syst Biol. 2013;9:704 10.1038/msb.2013.56 . 24189399 PMC4039373

[21] Hao N O’Shea EK. Signal-dependent dynamics of transcription factor translocation controls gene expression. Nat Struct Mol Biol. 2012;19(1):31–9. Epub 2011/12/20. 10.1038/nsmb.2192nsmb.2192 [pii] . 22179789 PMC3936303

[22] Daniels B. C., Ryu W. S., & Nemenman I. (2019). Automated, predictive, and interpretable inference of Caenorhabditis elegans escape dynamics. Proceedings of the National Academy of Sciences of the United States of America, 116(15), 7226–7231. 10.1073/pnas.1816531116 30902894 PMC6462057

[23] Munsky B., Li G., Fox Z. R., Shepherd D. P., & Neuert G. (2018). Distribution shapes govern the discovery of predictive models for gene regulation. Proceedings of the National Academy of Sciences of the United States of America, 115(29), 7533–7538. 10.1073/pnas.1804060115 29959206 PMC6055173

[24] Goentoro L, Shoval O, Kirschner MW, Alon U. The incoherent feedforward loop can provide fold-change detection in gene regulation. Molecular cell. 2009;36(5):894–9. Epub 2009/12/17. S1097-2765(09)00859-4 [pii]10.1016/j.molcel.2009.11.018. 10.1016/j.molcel.2009.11.018 20005851 PMC2896310

[25] Ma W, Trusina A, El-Samad H, Lim WA, Tang C. Defining network topologies that can achieve biochemical adaptation. Cell. 2009;138(4):760–73. Epub 2009/08/26. S0092-8674(09)00712-0 [pii]10.1016/j.cell.2009.06.013. 10.1016/j.cell.2009.06.013 19703401 PMC3068210

[26] Purvis JE, Karhohs KW, Mock C, Batchelor E, Loewer A, Lahav G. p53 dynamics control cell fate. Science. 2012;336(6087):1440–4. 10.1126/science.1218351 22700930 PMC4162876

[27] Alon U. (2007). An introduction to systems biology: Design principles of biological circuits. Boca Raton, FL: CRC Press/Taylor & Francis Group.

[28] Shellhammer JP, Pomeroy AE, Li Y, Dujmusic L, Elston TC, Hao N, et al Quantitative analysis of the yeast pheromone pathway. Yeast. 2019 10.1002/yea.3395 . 31022772 PMC6684483

[29] Hansen AS O’Shea EK. Encoding four gene expression programs in the activation dynamics of a single transcription factor. Curr Biol. 2016;26(7):R269–71. 10.1016/j.cub.2016.02.058 . 27046808

[30] Kawakami E, Singh VK, Matsubara K, Ishii T, Matsuoka Y, Hase T, et al Network analyses based on comprehensive molecular interaction maps reveal robust control structures in yeast stress response pathways. NPJ Syst Biol Appl. 2016;2:15018 10.1038/npjsba.2015.18 28725465 PMC5516916

[31] Goulev Y, Morlot S, Matifas A, Huang B, Molin M, Toledano MB, et al Nonlinear feedback drives homeostatic plasticity in H2O2 stress response. Elife. 2017;6 10.7554/eLife.23971 28418333 PMC5438251

[32] Selimkhanov J, Taylor B, Yao J, Pilko A, Albeck J, Hoffmann A, et al Systems biology. Accurate information transmission through dynamic biochemical signaling networks. Science. 2014;346(6215):1370–3. 10.1126/science.1254933 25504722 PMC4813785

[33] Handly LN, Yao J, Wollman R. Signal Transduction at the Single-Cell Level: Approaches to Study the Dynamic Nature of Signaling Networks. J Mol Biol. 2016;428(19):3669–82. 10.1016/j.jmb.2016.07.009 27430597 PMC5023475

[34] Spiller DG, Wood CD, Rand DA, White MR. Measurement of single-cell dynamics. Nature. 2010;465(7299):736–45. 10.1038/nature09232 . 20535203

[35] Wollman R. Robustness, Accuracy, and Cell State Heterogeneity in Biological Systems. Curr Opin Syst Biol. 2018;8:46–50. 10.1016/j.coisb.2017.11.009 29308439 PMC5752152

[36] Yuan GC, Cai L, Elowitz M, Enver T, Fan G, Guo G, et al Challenges and emerging directions in single-cell analysis. Genome Biol. 2017;18(1):84 10.1186/s13059-017-1218-y 28482897 PMC5421338

[37] Weinreb C, Wolock S, Tusi BK, Socolovsky M, Klein AM. Fundamental limits on dynamic inference from single-cell snapshots. Proceedings of the National Academy of Sciences of the United States of America. 2018;115(10):E2467–E76. 10.1073/pnas.1714723115 29463712 PMC5878004

[38] Dunlop M. J., Cox R. S. 3rd, Levine J. H., Murray R. M., & Elowitz M. B. (2008). Regulatory activity revealed by dynamic correlations in gene expression noise. Nature genetics, 40(12), 1493–1498. 10.1038/ng.281 19029898 PMC2829635

[39] Gillies T. E., Pargett M., Minguet M., Davies A. E., & Albeck J. G. (2017). Linear Integration of ERK Activity Predominates over Persistence Detection in Fra-1 Regulation. Cell systems, 5(6), 549–563.e5. 10.1016/j.cels.2017.10.019 29199017 PMC5746471

[40] Wilson M. Z., Ravindran P. T., Lim W. A., & Toettcher J. E. (2017). Tracing Information Flow from Erk to Target Gene Induction Reveals Mechanisms of Dynamic and Combinatorial Control. Molecular cell, 67(5), 757–769.e5. 10.1016/j.molcel.2017.07.016 28826673 PMC5591080

[41] Farkash-Amar S, Zimmer A, Eden E, Cohen A, Geva-Zatorsky N, Cohen L, et al Noise genetics: inferring protein function by correlating phenotype with protein levels and localization in individual human cells. PLoS Genet. 2014;10(3):e1004176 Epub 2014/03/08. 10.1371/journal.pgen.1004176 24603725 PMC3945223

[42] Kim Y, Han S, Choi S, Hwang D. Inference of dynamic networks using time-course data. Briefings in bioinformatics. 2014;15(2):212–28. 10.1093/bib/bbt028 . 23698724

[43] Loewer A, Batchelor E, Gaglia G, Lahav G. Basal dynamics of p53 reveal transcriptionally attenuated pulses in cycling cells. Cell. 2010;142(1):89–100. Epub 2010/07/06. S0092-8674(10)00567-2 [pii] PubMed Central PMCID: PMC3003696. 10.1016/j.cell.2010.05.031 20598361 PMC3003696

[44] Neuert G, Munsky B, Tan RZ, Teytelman L, Khammash M, van Oudenaarden A. Systematic identification of signal-activated stochastic gene regulation. Science. 2013;339(6119):584–7. Epub 2013/02/02. 10.1126/science.1231456 . 23372015 PMC3751578

