# Supplementary material for: Inferring the structures of signaling motifs from paired dynamic traces of single cells
Source: PLoS Comput Biol. 2021 Feb 4;17(2):e1008657. doi: 10.1371/journal.pcbi.1008657 (PMC7889133; doi:10.1371/journal.pcbi.1008657)
Supplement: S1 Text — (DOCX) [file pcbi.1008657.s001.docx]

Supplemental Information

Inferring the structures of signaling motifs from paired dynamic traces of single cells

Raymond A. Haggerty^1,2,3^ and Jeremy E. Purvis^1,2,3,4,^*

^1^Department of Genetics, University of North Carolina at Chapel Hill, North Carolina, USA
^2^Computational Medicine Program, University of North Carolina at Chapel Hill, North Carolina, USA
^3^Curriculum for Bioinformatics and Computational Biology, University of North Carolina at Chapel Hill, North Carolina, USA
^4^Lineberger Comprehensive Cancer Center, University of North Carolina at Chapel Hill, North Carolina, USA

*Corresponding Author:
Jeremy Purvis
Mary Ellen Jones Building 11018C, CB#7488
116 Manning Drive
Chapel Hill, NC 27599-7488
jeremy_purvis@med.unc.edu

***Materials and Methods:***

**Data Preparation:**

Raw data was arrays of average arbitrary fluorescent units for each cell. The first X timepoints prior to the stimulation of the cells were used as the basal levels, and the average of those timepoints was used to find the foldchange of the cells by setting those to one. Implementation details can be found in dataprep.m

**Creation of Synthetic Data Set:**

The synthetic data set was created by starting with a set of 50 identical step functions of 1000 seconds with a fold change of 2, stepping up at 200 seconds and back down at 800 seconds. We then adjusted it so that each “cell” had a different start and stop to the step function as well as a different amplitude. We then used the ODE equations for the incoherent feed forward loop, as described below. Each “cell” was given the same parameter set of 0.2 for a, 1.5 for K and 2 for n.

The additional three synthetic data sets were created in a similar fashion, with different hill equations and parameter sets to make them behave in their characteristic fashion.

**Parameter Bounds:**

a is bounded between 0 and 1 because the degradation rate must be a fraction between 0 and 1.

K, which represents the half max of the fold change is bounded between 0 and 100. This covers a broad biological range.

n is bounded between 1 and 4. The hill constant is usually between 1 and 4 for cooperative binding.

**Practical Limits of MISC Testing:**

In our manuscript we made the decision to test four known motifs thoroughly with MISC to characterize how well MISC was able to predict the correct response. We chose these four motifs because we wanted well characterized motifs that we know have a biological function to test the robustness of MISC.

In order to use a motif as a known motif to test MISC’s ability with, we need to first make sure that the motif is correctly parameterized to produce its characteristic behavior. The characteristic behavior of many motifs is unknown, making it difficult to parameterize them in a way that would give us meaningful results about MISC’s ability. This is not a problem for recognizing motifs from biological data, as biological motifs are inherently parameterized to have their characteristic behavior.

In addition, we note that there are many motifs which produce the same biological function with different motif structures (e.g. Incoherent Feed Forward Loops). MISC is expected to perform equally well on these structures, making testing on them redundant.

Finally, there are time considerations as to why we didn’t test all 402 motifs. One of the limitations of MISC is that it does take a considerable time to run and parameterize the full complement of 402 motifs for each motif tested, and it was therefore infeasible to run all 402 motifs for testing in a reasonable time.

***Coding:***

**Code Implementation:**

All code was written in MATLAB 2018a Please see the included files dY_hill.m and dZ_hill.m to see the implementation of the full equations.

**Estimation of time to run:**

MISC takes approximately 30 hours to run on the UNC longleaf server, on a single compute node with 24 physical cores, 2.50 GHz Intel processors, 30M cache (Model E5-2680 v3), 256-GB RAM, and 2x10Gbps NIC. Additional analysis is done on a local computer in minutes.

***ODE Derivation:***

**Hill Equations:**

Hill equations are widely used in biochemistry, as they provide a more accurate model of biological processes than using simple zeroth or first order kinetics. The following are the typical forms of the hill equations for activation and repression:

Activation:

$$\frac{dZ}{dt}=\beta\frac{X^{n}}{K_{XZ}^{n}+X^{n}}-\alpha Z$$

Repression:

$$\frac{dZ}{dt}=\beta\frac{1}{1+\left( \frac{Y}{K_{YZ}} \right)^{n}}-\alpha Z$$

Where:

$\beta=$Maximal production rate of promoter

$K_{XZ}=$ Halfway activation point of Z by X

$K_{YZ}=$Halfway repression point of Z by Y

$n=$Hill Coefficient, cooperativity

$\alpha=$Degradation rate of protein

When multiple factors are affecting one node, the core of the equations are multiplied together into a compound equation to get the full effect, while the production rate and degradation rate remain the same. As an example, if Z is activated by X and repressed by Y, the total equation of the effect on Z would be:

$$\frac{dZ}{dt}=\beta\left( \frac{X^{n}}{K_{XZ}^{n}+X^{n}} \right)\left( \frac{1}{1+\left( \frac{Y}{K_{YZ}} \right)^{n}} \right)-\alpha Z$$

**Autogeneration of compound equations:**

Given an array $g$ of the connections between each node to Y and Z, with one indicating activation, negative one indicating repression and zero indicating no connection, we can autogenerate the compound equations using $g$ to determine which equations to include.

$$\frac{dY}{dt}=\left( \beta\left( \left( eq_{activator} \right)^{{g(1)}^{2}\left( \frac{g(1)+1}{2} \right)}\left( \frac{1}{eq_{repressor}} \right)^{{g(1)}^{2}\left( \frac{g(1)-1}{2} \right)} \right)*\left( \left( eq_{activator} \right)^{{g(2)}^{2}\left( \frac{g(2)+1}{2} \right)}\left( \frac{1}{eq_{repressor}} \right)^{{g(2)}^{2}\left( \frac{g(2)-1}{2} \right)} \right)*\left( \left( eq_{activator} \right)^{{g(3)}^{2}\left( \frac{g(3)+1}{2} \right)}\left( \frac{1}{eq_{repressor}} \right)^{{g(3)}^{2}\left( \frac{g(3)-1}{2} \right)} \right) \right)\max\left( {g(1:3)}^{2} \right)-\alpha Y$$

$$\frac{dZ}{dt}=\left( \beta\left( \left( eq_{activator} \right)^{{g(4)}^{2}\left( \frac{g(4)+1}{2} \right)}\left( \frac{1}{eq_{repressor}} \right)^{{g(4)}^{2}\left( \frac{g(4)-1}{2} \right)} \right)*\left( \left( eq_{activator} \right)^{{g(5)}^{2}\left( \frac{g(5)+1}{2} \right)}\left( \frac{1}{eq_{repressor}} \right)^{{g(5)}^{2}\left( \frac{g(5)-1}{2} \right)} \right)*\left( \left( eq_{activator} \right)^{{g(6)}^{2}\left( \frac{g(6)+1}{2} \right)}\left( \frac{1}{eq_{repressor}} \right)^{{g(6)}^{2}\left( \frac{g(6)-1}{2} \right)} \right) \right)\max\left( {g(4:6)}^{2} \right)-\alpha Z$$

Where:

$g$ = [x->y, y->y, z->y, x->z, y->z, z->z]

With:

0 = no connection

1 = activation

-1 = repression

And:

eq_activator_  = equation for activation of the node

eq_repressor_ = equation for the repression of the node

When the connection is 1, the activator equation is selected for that connection. If it is -1, the repressor equation is selected. If it is 0, the connection becomes one. If all connections are zero, then the selection of the maximum for the connections ensures that the total effect is zero.

**Fold Change Equations:**

Hill equations are in units of concentration. However, when working with microscopy images, we are working in the realm of arbitrary fluorescent units and are unable to translate those into units of absolute concentration. We are able to get fold changes from a basal steady state. Therefore, it is necessary to get the hill equations into a form where they will work on fold change rather than absolute concentration.

At steady state, the change in Z is zero. As stated above the change in Z is as follows:

$$\Delta Z=\beta f\left( X \right)g\left( Y \right)-\alpha Z$$

$$0=\beta f\left( X_{st} \right)g\left( Y_{st} \right)-\alpha Z_{st}$$

Solve for the steady state of Z:

$$\alpha Z_{st}=\beta f\left( X_{st} \right)\left( Y_{st} \right)$$

$$Z_{st}=\frac{\beta}{\alpha}f\left( X_{st} \right)g\left( Y_{st} \right)$$

The fold change is equal to the current state of Z divided by the steady state of Z:

$$Z_{fc}=\frac{Z}{Z_{st}}$$

$$\frac{\Delta Z}{Z_{st}}=\frac{\beta f\left( X \right)g\left( Y \right)-\alpha Z}{\frac{\beta}{\alpha}f\left( X_{st} \right)g\left( Y_{st} \right)}$$

Simplify:

$$\frac{\Delta Z}{Z_{st}}=\frac{\left( \frac{\alpha}{\beta} \right)\beta f\left( X \right)g\left( Y \right)}{\left( \frac{\alpha}{\beta} \right)\frac{\beta}{\alpha}f\left( X_{st} \right)g(Y_{st})}-\frac{\alpha Z}{\frac{\beta}{\alpha}f\left( X_{st} \right)g\left( Y_{st} \right)}$$

$$\frac{\Delta Z}{Z_{st}}= \frac{\alpha f\left( X \right)g\left( Y \right)}{f\left( X_{st} \right)g\left( Y_{st} \right)}-\alpha\frac{Z}{Z_{st}}$$

$$\Delta Z_{fc}=\frac{\alpha f\left( X \right)g\left( Y \right)}{f\left( X_{st} \right)g\left( Y_{st} \right)}-\alpha Z_{fc}$$

**Full Equations:**

Putting together the fold change equations with the autogeneration of the compound equations, we get the following full equations:

$$\frac{dY}{dt}=a\left( 1 \right)*\left( \left( \left( \frac{\left( \frac{x^{n\left( 1 \right)}}{k\left( 1 \right)^{n\left( 1 \right)}+x^{n\left( 1 \right)}} \right)^{g\left( 1 \right)^{2}*\left( \frac{g\left( 1 \right)+1}{2} \right)}*\left( \frac{k\left( 1 \right)^{n\left( 1 \right)}+x^{n\left( 1 \right)}}{k\left( 1 \right)^{n\left( 1 \right)}} \right)^{g\left( 1 \right)^{2}*\left( \frac{g\left( 1 \right)-1}{2} \right)}}{\left( \frac{1}{k\left( 1 \right)^{n\left( 1 \right)}+1} \right)^{g\left( 1 \right)^{2}*\left( \frac{g\left( 1 \right)+1}{2} \right)}*\left( \frac{k\left( 1 \right)^{n\left( 1 \right)}+1}{k\left( 1 \right)^{n\left( 1 \right)}} \right)^{g\left( 1 \right)^{2}*\left( \frac{g\left( 1 \right)-1}{2} \right)}} \right)*\left( \frac{\left( \frac{y^{n\left( 2 \right)}}{k\left( 2 \right)^{n\left( 2 \right)}+y^{n\left( 2 \right)}} \right)^{g\left( 2 \right)^{2}*\left( \frac{g\left( 2 \right)+1}{2} \right)}*\left( \frac{k\left( 2 \right)^{n\left( 2 \right)}+y^{n\left( 2 \right)}}{k\left( 2 \right)^{n\left( 2 \right)}} \right)^{g\left( 2 \right)^{2}*\left( \frac{g\left( 2 \right)-2}{2} \right)}}{\left( \frac{1}{k\left( 2 \right)^{n\left( 2 \right)}+1} \right)^{g\left( 2 \right)^{2}*\left( \frac{g\left( 2 \right)+1}{2} \right)}*\left( \frac{k\left( 2 \right)^{n\left( 2 \right)}+1}{k\left( 1 \right)^{n\left( 2 \right)}} \right)^{g\left( 2 \right)^{2}*\left( \frac{g\left( 2 \right)-1}{2} \right)}} \right)*\left( \frac{\left( \frac{z^{n\left( 3 \right)}}{k\left( 3 \right)^{n\left( 3 \right)}+z^{n\left( 3 \right)}} \right)^{g\left( 3 \right)^{2}*\left( \frac{g\left( 3 \right)+1}{2} \right)}*\left( \frac{k\left( 3 \right)^{n\left( 3 \right)}+z^{n\left( 3 \right)}}{k\left( 3 \right)^{n\left( 3 \right)}} \right)^{g\left( 3 \right)^{2}*\left( \frac{g\left( 3 \right)-1}{2} \right)}}{\left( \frac{1}{k\left( 3 \right)^{n\left( 3 \right)}+1} \right)^{g\left( 3 \right)^{2}*\left( \frac{g\left( 3 \right)+1}{2} \right)}*\left( \frac{k\left( 3 \right)^{n\left( 3 \right)}+1}{k\left( 3 \right)^{n\left( 3 \right)}} \right)^{g\left( 3 \right)^{2}*\left( \frac{g\left( 3 \right)-1}{2} \right)}} \right) \right)-y \right)$$

$$\frac{dZ}{dt}=a\left( 2 \right)*\left( \left( \left( \frac{\left( \frac{x^{n\left( 4 \right)}}{k\left( 4 \right)^{n\left( 4 \right)}+x^{n\left( 4 \right)}} \right)^{g\left( 4 \right)^{2}*\left( \frac{g\left( 4 \right)+1}{2} \right)}*\left( \frac{k\left( 4 \right)^{n\left( 4 \right)}+x^{n\left( 4 \right)}}{k\left( 4 \right)^{n\left( 4 \right)}} \right)^{g\left( 4 \right)^{2}*\left( \frac{g\left( 4 \right)-1}{2} \right)}}{\left( \frac{1}{k\left( 4 \right)^{n\left( 4 \right)}+1} \right)^{g\left( 4 \right)^{2}*\left( \frac{g\left( 4 \right)+1}{2} \right)}*\left( \frac{k\left( 4 \right)^{n\left( 4 \right)}+1}{k\left( 4 \right)^{n\left( 4 \right)}} \right)^{g\left( 4 \right)^{2}*\left( \frac{g\left( 4 \right)-1}{2} \right)}} \right)*\left( \frac{\left( \frac{y^{n\left( 5 \right)}}{k\left( 5 \right)^{n\left( 5 \right)}+y^{n\left( 5 \right)}} \right)^{g\left( 5 \right)^{2}*\left( \frac{g\left( 5 \right)+1}{2} \right)}*\left( \frac{k\left( 5 \right)^{n\left( 5 \right)}+y^{n\left( 5 \right)}}{k\left( 5 \right)^{n\left( 5 \right)}} \right)^{g\left( 5 \right)^{2}*\left( \frac{g\left( 5 \right)-2}{2} \right)}}{\left( \frac{1}{k\left( 5 \right)^{n\left( 5 \right)}+1} \right)^{g\left( 5 \right)^{2}*\left( \frac{g\left( 5 \right)+1}{2} \right)}*\left( \frac{k\left( 5 \right)^{n\left( 5 \right)}+1}{k\left( 5 \right)^{n\left( 5 \right)}} \right)^{g\left( 5 \right)^{2}*\left( \frac{g\left( 5 \right)-1}{2} \right)}} \right)*\left( \frac{\left( \frac{z^{n\left( 6 \right)}}{k\left( 6 \right)^{n\left( 6 \right)}+z^{n\left( 6 \right)}} \right)^{g\left( 6 \right)^{2}*\left( \frac{g\left( 6 \right)+1}{2} \right)}*\left( \frac{k\left( 6 \right)^{n\left( 6 \right)}+z^{n\left( 6 \right)}}{k\left( 6 \right)^{n\left( 6 \right)}} \right)^{g\left( 6 \right)^{2}*\left( \frac{g\left( 6 \right)-1}{2} \right)}}{\left( \frac{1}{k\left( 6 \right)^{n\left( 6 \right)}+1} \right)^{g\left( 6 \right)^{2}*\left( \frac{g\left( 6 \right)+1}{2} \right)}*\left( \frac{k\left( 6 \right)^{n\left( 6 \right)}+1}{k\left( 6 \right)^{n\left( 6 \right)}} \right)^{g\left( 6 \right)^{2}*\left( \frac{g\left( 6 \right)-1}{2} \right)}} \right) \right)-z \right)$$

Where:

$a$ = vector with the degradation rates of y and z

$k$ = vector of the halfway activation points

$n$ = vector of the cooperativity

$g$ = vector of connectivity

$x$ = vector of factor x

$y$= vector of factor y

$z$ = vector of factor z

***Hyperparameter Selection:***

**Table A. Selection of Top Motifs:**

We used several methods to determine the top motifs and then determined the average distance to the correct IFFL motif. Although the methods are all similar, the elbow method at 10% was the best performing, and this was the method we used in all of our selections.

| **n metric** | **avg distance** |
| --- | --- |
| **top 100** | 1.8847 |
| **top 1000** | 1.8552 |
| **elbow (1%)** | 1.8301 |
| **elbow (10%)** | 1.7849 |
| **top 0.01%** | 1.8583 |
| **top 0.1%** | 1.878 |

**Figure A. Selection of number of parameters:**

We tried several different sizes of numbers of parameters to determine the point at where adding more parameters was no longer beneficial. 100,000 parameters performed only marginally better than 40,000. Because we the graph began to level out at 40,000 and we had already invested the computational time in running the 40,000 parameter sets for all of our data, we used 40,000 parameters.

**
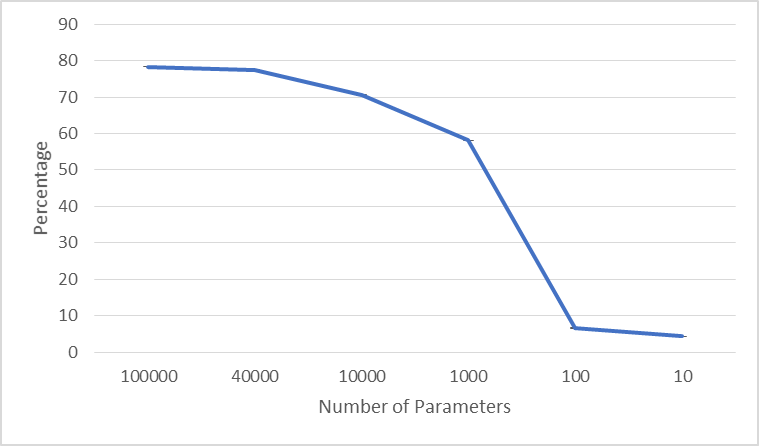
**

**Tutorial.** A tutorial file is included as Supporting Information
