# Supplementary material for: Inferring the structures of signaling motifs from paired dynamic traces of single cells
Source: PLoS Comput Biol. 2021 Feb 4;17(2):e1008657. doi: 10.1371/journal.pcbi.1008657 (PMC7889133; doi:10.1371/journal.pcbi.1008657)
Supplement: S1 Tutorial — (PDF) [file pcbi.1008657.s002.pdf]

How to run MISC:

Download files from:
https://figshare.com/projects/Inferring_the_structures_of_signaling_motifs_from_paired_dynamic_traces_of_single_cells/73275
You will need to download all files in “Code Repository” and “Figure 3 Reproduction Files”
This tutorial will cover how to reproduce figure 3 D-F.

From inside the code repository, run the following command in MATLAB:
```
>> [sMSE, params_out, graphs] = MISC(datafile,numParam)
```
Where datafile is the location of IN_IFFL.mat and numParam is the number of parameter sets you want to sample (40,000 in the paper). This is a time and resource intensive step, so you may desire to skip it. For the paper, this was run on UNC’s compute cluster.

If you do, you may simply load both IN_IFFL.mat and OUT_IFFL.mat into your workspace. OUT_IFFL.mat contains the outputs of running MISC on IN_IFFL.mat with 40,000 parameters.

From inside the code repository, run the following command in MATLAB:
```
>> getgraphsnew;
```

The resultant figure 1 is equivalent to the inset of figure 3D, figure 2 is equivalent to figure 3E and figure 3 is equivalent to figure 3F.
